# Enhanced magnetic thermal ablation combined with immunotherapy for hepatocellular carcinoma using engineering microspheres

**DOI:** 10.1016/j.mtbio.2025.102597

**Published:** 2025-11-27

**Authors:** Zepeng Yu, Yaping He, Mengmeng Wang, Jiaofeng Shen, Di Wang, Andong Yu, Jun Gu, Zhihui Hong, Zhijun Pei, Xingwei Sun

**Affiliations:** a Department of Interventional Radiology, The Second Affiliated Hospital of Soochow University, Suzhou, 215004, China; b Center for Medical Ultrasound, The Affiliated Suzhou Hospital of Nanjing Medical University, Suzhou Municipal Hospital, Gusu School, Nanjing Medical University, Suzhou, 215001, Jiangsu Province, China; c Department of Radiotherapy & Oncology, The Second Affiliated Hospital of Soochow University, Suzhou, 215004, China; d Department of Pharmacy, The Second Affiliated Hospital of Soochow University, Suzhou, 215004, China; e Department of Oncology, The Second Affiliated Hospital of Soochow University, Suzhou, 215004, China; f Department of Nuclear Medicine, The Second Affiliated Hospital of Soochow University, Suzhou, 215004, China

**Keywords:** Hepatocellular carcinoma, Magnetic thermal ablation, Microspheres, PD-1 monoclonal antibodies, Transcatheter arterial embolization

## Abstract

Hepatocellular carcinoma (HCC) remains a formidable challenge in oncology, characterized by high metastatic potential and recurrence rates. Traditional microwave ablation technique faces limitations due to operator dependency and potential complications. To address these challenges, we developed micron-sized polyacrylamide-iron carbonyl magnetic hyperthermia microspheres (PAM@Fe(CO)_5_ MSs) for non-invasive magnetic thermal ablation (MTA). These microspheres, synthesized via the SPG membrane emulsification technique, exhibit commendable biocompatibility, size stability, and robust magnetic thermal effects. When combined with PD-1 monoclonal antibody immunotherapy, PAM@Fe(CO)_5_ MSs demonstrated significant synergistic effects, leading to the markedly curtailed growth of metastatic tumors in mouse models. Further, in a rabbit orthotopic liver tumor model, MTA using PAM@Fe(CO)_5_ MSs showcased excellent safety and efficacy, with minimal impact on liver function and no observable toxicity in critical organs. This innovative approach not only enhances anti-tumor efficacy by activating the host's T-cell immune response but also overcomes the immune-suppressive tumor microenvironment. Our findings suggest that combining MTA with immunotherapy may offer a viable treatment approach for HCC and possibly other solid tumors, paving the way for safer and more effective clinical applications.

## Graphical abstract

**Figure:**
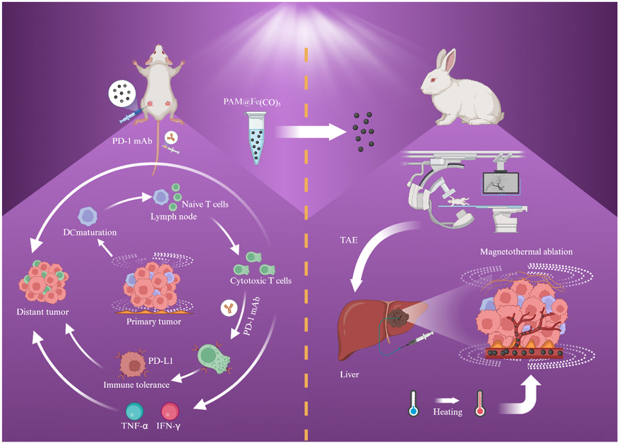

## 1 Introduction

Hepatocellular carcinoma (HCC) is the most prevalent form of liver cancer, known for its high rates of metastasis and recurrence, which pose significant challenges to effective clinical management [1,2]. In recent years, image-guided percutaneous ablation methods, particularly microwave ablation (MWA), have emerged as vital treatment options for HCC [3,4]. These techniques work by delivering thermal energy directly to tumor tissue, inducing cell death [5]. However, the efficacy of MWA is often hindered by operator dependency during the puncture process, which can lead to complications including damage to adjacent organs and unintended tumor seeding along the puncture tract [6,7]. Furthermore, the presence of larger or multiple tumor lesions requires multiple punctures, increasing the risk of adverse effects [8]. This underlines the pressing need for safer and more effective ablation strategies.

Magnetic hyperthermia (MHT) represents a promising alternative therapy that employs heat generated by magnetic materials in response to a high-frequency alternating magnetic field (AMF) [[9], [10], [11], [12]]. MHT offers several advantages, such as enhanced precision in targeting tumors and the capability to address limitations associated with tissue depth [13,14]. However, existing research predominantly centers on nano-sized magnetic particles or macro-scale materials that either have limited tumor accumulation or necessitate invasive implantation procedures [15,16]. To surmount these obstacles, we developed micron-sized iron carbonyl (Fe(CO)_5_) magnetic hyperthermia microspheres (PAM@Fe(CO)_5_ MSs) and utilized transcatheter arterial embolization (TAE) for targeted delivery to the blood supply of liver tumors. This innovative strategy effectively restricts blood flow to tumors while improving the accumulation of PAM@Fe(CO)_5_ MSs, thereby facilitating non-invasive magnetic thermal ablation (MTA). Despite these advancements, the immune-suppressive tumor microenvironment poses a significant hurdle, making it difficult to prevent recurrence and metastasis following MTA.

Recent advances in immunotherapy have yielded encouraging results across various tumor types, including HCC, by effectively suppressing tumor metastasis and recurrence [17,18]. The combination of MTA with immunotherapy holds great promise as a comprehensive treatment strategy for tumors [[19], [20], [21]]. Notably, the development of novel therapeutic agents further expands the options for improving the efficacy of treatment modalities for HCC. One innovative approach demonstrates the use of self-fueling ferroptosis-inducing microreactors based on pH-responsive Lipiodol Pickering emulsions. These formulations enhance the stability of chemotherapeutic agents, reducing systemic toxicity while effectively inducing cancer cell death through ferroptosis upon transarterial embolization [22]. Such microreactors exemplify how improving drug delivery systems can lead to superior therapeutic outcomes.

Another relevant study involves procoagulant calcium carbonate (CaCO_3_)-embedded embolic MSs that potentiate TAE of HCC. These MSs are designed to occlude tumor vasculature effectively and neutralize the acidic tumor microenvironment, thus enhancing tumor suppression and reversing immunosuppression. This biocompatibility and efficacy position them as promising candidates to elevate conventional TAE treatments for HCC [23].

**Scheme 1:**
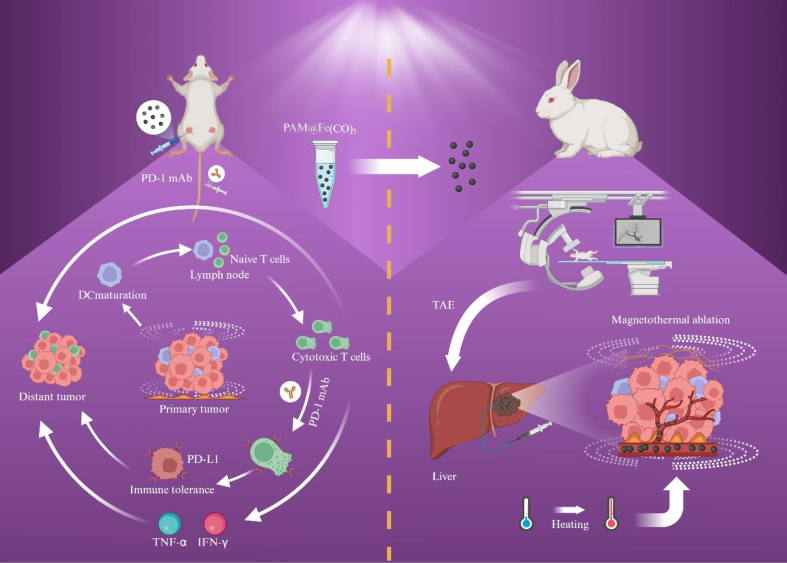
Schematic illustration of the preparation of PAM@Fe(CO)_5_ MSs utilizing SPG membrane emulsification technology, which function as MTA agents for combined MTA-immunotherapy in mouse xenograft models. Additionally, it highlights the application of TAE-MTA in the treatment of rabbit orthotopic VX2 liver tumors.

In this study, we developed polyacrylamide-iron carbonyl magnetic hyperthermia microspheres (PAM@Fe(CO)_5_ MSs), which exhibited excellent biocompatibility, size stability, and effective heat induction through the SPG membrane emulsification technique. These MSs exhibited robust and consistent magnetic thermal effects in the presence of an AMF, successfully inducing tumor cell death while simultaneously stimulating the host's anti-tumor immune response. The combination of PAM@Fe(CO)_5_ MSs with PD-1 monoclonal antibody (PD-1 mAb) immunotherapy resulted in complete ablation of primary HCC tumors and significantly inhibited the growth of metastatic tumors, highlighting the substantial synergistic effects of this treatment. Our approach utilizing PAM@Fe(CO)_5_ MSs for MTA demonstrated enhanced safety and therapeutic efficacy in the rabbit tumor model, reinforcing its potential for future clinical applications (see Scheme 1).

## 2 Methods and materials

### 2.1 Synthesis of PAM@Fe(CO)_5_ MSs

PAM@Fe(CO)_5_ MSs were synthesized utilizing the SPG membrane emulsification technique, with the content of Fe(CO)_5_ adjusted to optimize the magnetic thermal properties. The specific procedure involved thoroughly mixing a certain amount of nano-sized Fe(CO)_5_ dispersion, polyethylene glycol (PEG), and polyacrylamide (PAM) with 10 mL of SDS aqueous solution. This homogeneous mixture was subsequently fed into the SPG membrane emulsification system, where the emulsification pore size was set to 20 μm and the pressure was maintained at 0.5 MPa. As a result of this process, PAM@Fe(CO)_5_ MSs with diameters of approximately 50 μm were produced, featuring varying concentrations of Fe(CO)_5_ at 5 %, 15 %, and 25 %. Commercial nano-sized Fe(CO)_5_ was purchased from Jiangsu Zhichuan Technology Co., Ltd., which is a dietary iron supplement with guaranteed biological safety.

### 2.2 Evaluation of magnetic thermal effect and characterization of PAM@Fe(CO)_5_ MSs

AMF was generated using a high-frequency induction heating device (SPG-10AB-11) with two heating coils (5.0 cm and 15.0 cm in diameter). Characterization of PAM@Fe(CO)_5_ MSs was carried out via scanning electron microscopy (SEM, ZEISS) to capture micrographs, and the diameters of MSs in different groups were measured using ImageJ software to create size distribution graphs. Additionally, thermal analysis of the prepared materials was conducted using a Diamond thermal analyzer from PerkinElmer.

### 2.3 Cell experiments

For in vitro MHT studies, various cell types (L929, H22, 4T1, and VX2) were exposed to PAM@Fe(CO)_5_ MSs in AMF for 5 min. Treatment temperatures were set at 42 °C (f_appl_ = 250 kHz, H_appl_ = 4.5 × 10^3^ A m^−1^), 47 °C (f_appl_ = 250 kHz, H_appl_ = 5.5 × 10^3^ A m^−1^), and 52 °C (f_appl_ = 250 kHz, H_appl_ = 6.5 × 10^3^ A m^−1^). Prior to testing, cells were seeded in culture dishes, and upon reaching approximately 80 % confluence, they were directly treated with UV-sterilized PAM@Fe(CO)_5_ MSs under AMF. The medium temperature was monitored using a thermal imager, and dishes were gently shaken to ensure uniform heating of the cells. In the control group, these cells were maintained under identical culture conditions but did not receive any treatment with PAM@Fe(CO)_5_ MSs or exposure to an AMF.

### 2.4 Pathological sample acquisition from liver cancer patients

To further investigate the impact of microwave ablation (MWA) treatment on the tumor microenvironment in liver cancer patients, relevant pathological samples were obtained from the Department of Pathology. Surgical specimens and biopsy samples underwent routine pathological processing, including fixation, dehydration, and embedding, followed by sectioning into 4-μm thick slices. Immunohistochemical staining was performed on the slices to assess T cell infiltration. Prior to the study, informed written consent was secured from all participants or their next of kin. The research adhered to the principles outlined in the Declaration of Helsinki. The Ethics Committee of Our Hospital proved all studies (K-2023-050-H01).

### 2.5 In vivo experiments

Each mouse received an inoculation of 2 × 10^6^ H22 cells in the left flank to create a primary tumor and 0.5 × 10^6^ H22 cells in the right flank to simulate distant metastasis. The mice were randomly divided into four groups: PD-1 mAb group (200 μg), MTA group (f_appl_ = 250 kHz, H_appl_ = 10.0 kA m^−1^), MTA + PD-1 mAb group (200 μg) and control group. Mice in the third and fourth groups received injections of PAM@Fe(CO)_5_ MSs into the left tumor and underwent MTA for complete ablation.

Mice in the fourth group were given intravenous injections of 200 μg PD-1 mAb following MTA treatment. In contrast, mice in the second group received only three intravenous doses of PD-1 mAb. Mice in the first and second groups underwent complete surgical resection of the left primary tumor, while those in the third and fourth groups received complete ablation using MTA. Mice in the control group(the first group)did not receive any other treatment, meaning they were not subjected to MTA, did not receive PAM@Fe(CO)_5_ MSs injections, and did not receive PD-1 mAb. The survival times of mice in each group were documented and analyzed (n = 7).

### 2.6 Immune assessment

The right tumor and nearby lymph nodes (n = 4) were collected from the mice the day after treatment completion. The tumor tissues were cut into small pieces and digested to create a single-cell suspension. Subsequently, the cells were stained with antibodies. A similar procedure was applied to the adjacent lymph nodes. Immunohistochemical experiments using PD-L1 mAb were conducted, along with immunofluorescence experiments using CD4 and CD8 monoclonal antibodies. All flow cytometry antibodies used and their catalog numbers are listed in Supplementary Table 1. The operational details of flow cytometry can refer to our previous research [24]. The relative mRNA expression levels of PD-L1, IFN-γ, and TNF-α were assessed using PCR kits. The primer sequences are listed in Supplementary Table 2.

### 2.7 In vivo interventional embolization therapy

Male New Zealand white rabbits, each weighing 4 kg, were chosen for this study. VX2 cells were cultured and injected into the thigh muscle tissue of the New Zealand white rabbit. After a two-week period, tumor tissue was excised and sliced into approximately 1 mm^3^ blocks. Under ultrasound guidance, issue from the VX2 tumor was subsequently injected into the left lobe of the rabbit's liver. About 12 days later, CT scans were conducted to verify tumor development. Following this, the rabbits were assigned to three groups (n = 3): PAM@Fe(CO)_5_ MSs group (1 mL), and PAM@Fe(CO)_5_ MSs + AMF group (MTA group, f_appl_ = 250 kHz, H_appl_ = 10.0 kA m^−1^) and control group. Using the Seldinger technique, the femoral artery was punctured, and a 2.7F microcatheter was superselected to reach the tumor-feeding artery. Angiography confirmed the identification of the tumor artery under DSA (Siemens) guidance, and a slow infusion of PAM@Fe(CO)_5_ MSs suspension was administered. Real-time angiography was conducted to verify the distribution of the embolic agents and the resulting vascular occlusion, ensuring that the embolic agents did not reflux into non-target vessels. Following TAE, the rabbits in the MTA treatment group underwent MTA therapy. Upon initiation of MTA, infrared thermal imaging indicated a gradual increase in tumor temperature, which stabilized at approximately 70 °C for 5 min. Rabbits in the control group did not receive any treatment involving PAM@Fe(CO)_5_ MSs or AMF application. They underwent the same surgical procedures as the other groups, including catheterization via femoral artery puncture into the left hepatic artery and visualization of tumor-supplying blood vessels under DSA guidance, but no MSs were embolized into the tumor site.

Liver function indicators were monitored every four days. After one week of treatment, ^18^F-fluorodeoxyglucose (^18^F-FDG) positron emission tomography (PET, GE Discovery MI) imaging was conducted to assess the biological activity of tumors in each group. A 4 kg tumor-bearing rabbit was administered 0.5 mCi of ^18^F-FDG via ear vein injection, and PET imaging was performed 30 min later. Finally, the tumor-bearing rabbits were euthanized, and tumor tissues and organs were collected for histopathological analysis using hematoxylin and eosin (H&E) staining.

### 2.8 Statistical analysis

The data are expressed as mean ± SD, and ANOVA was conducted. A p-value of less than 0.05 (marked as ∗) was deemed statistically significant, while p-values below 0.01 (marked as ∗∗) and 0.001 (marked as ∗∗∗) were regarded as reflecting highly significant differences.

## 3 Results and discussion

### 3.1 Preparation and characterization of PAM@Fe(CO)_5_ multifunctional MSs

**Fig. 1:**
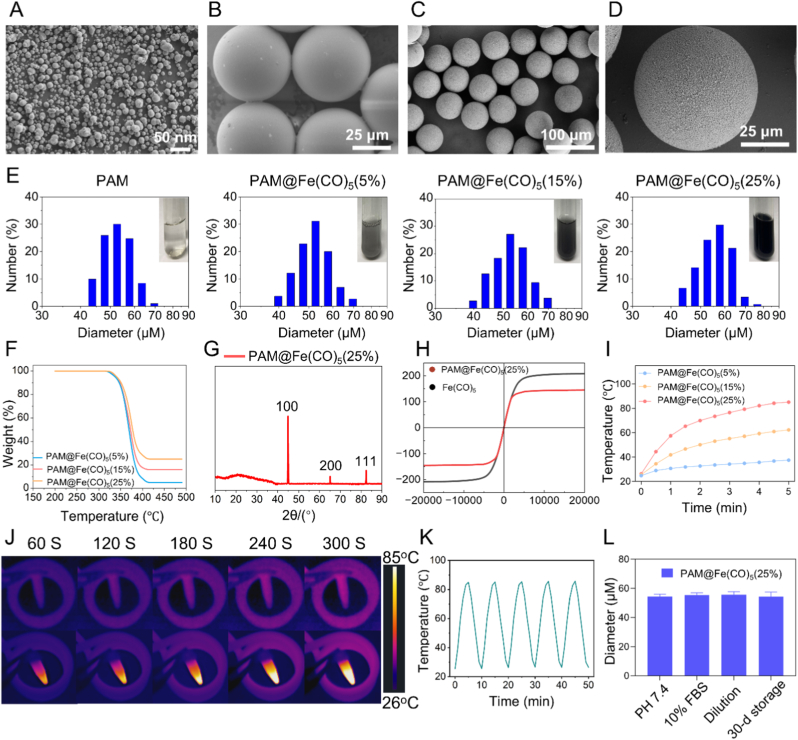
**Characterization results of PAM@Fe(CO)_5_ Microspheres (MSs)**. (A) Scanning electron microscopy (SEM) image of nano-sized Fe(CO)_5_. Scale bar = 50 nm (B) SEM image of micron-sized polyacrylamide (PAM). Scale bar = 25 μm. (C&D) SEM images showing the microstructure of PAM@Fe(CO)_5_ MSs, which have an average diameter of approximately 50 μm. Scale bar = 25 μm. (E) Showing the size distribution of PAM@Fe(CO)_5_ MSs with different Fe(CO)_5_ contents. Scale bar = 50 μm. (F) Showing the thermogravimetric analysis curves of PAM@Fe(CO)_5_ MSs with different Fe(CO)_5_ contents. (G) X-ray diffraction (XRD) pattern of PAM@Fe(CO)_5_ MSs containing 25 % Fe(CO)_5_. (H) Hysteresis loop of PAM@Fe(CO)_5_ MSs containing 25 % Fe(CO)_5_. (I) Displaying the heating temperature curves of PAM@Fe(CO)_5_ MSs containing 5 %, 15 %, and 25 % Fe(CO)_5_ over 5 min. (J) Temperature variation of PAM@Fe(CO)_5_ MSs containing 25 % Fe(CO)_5_ under alternating magnetic field (f_appl_ = 500 kHz, H_appl_ = 5.0 kA m^−1^) after 5 min of heating. (K) Repeated heating curves of PAM@Fe(CO)_5_ MSs with 25 % Fe(CO)_5_ over five cycles of alternating magnetic field. (L) In vitro stability assessment results of PAM@Fe(CO)_5_ MSs.

We successfully synthesized PAM@Fe(CO)_5_ MSs by integrating nanoscale Fe(CO)_5_ MSs (Fig. 1A) with micro-sized PAM MSs (Fig. 1B). The final PAM@Fe(CO)_5_ MSs measured approximately 50 μm in diameter and were produced through the SPG membrane emulsification technique (Fig. 1C and D). 50 μm PAM@Fe(CO)_5_ MSs were based on a thorough consideration of the particle size for embolic microspheres in clinical applications. Existing literature reports that the size of MSs significantly affects their distribution and embolic efficacy in vivo. MSs with a diameter of 50 μm are less likely to pass through capillaries, thereby facilitating complete embolization of tumor-supplying arteries and enhancing accumulation at tumor sites. In contrast, smaller MSs (e.g., diameters of 4–12 μm) can easily enter the venous circulation through capillaries, leading to safety concerns [[25], [26], [27]].

The SPG membrane emulsification technique offers notable advantages, particularly its ability to generate uniform and monodispersed droplets on a large scale. This method facilitates the flexible manipulation of monomer ratios, allowing for tailored properties in the final product. We achieved PAM@Fe(CO)_5_ MSs with varying Fe(CO)_5_ loadings of 5 %, 15 %, and 25 %, which resulted in MSs with consistent morphology and uniform distribution (Fig. 1E). However, it was observed that when the loading of Fe(CO)_5_ exceeded 30 %, we encountered significant challenges during arterial embolization using the catheter, resulting in blockages. These challenges manifested as blockages, likely due to the increased density of the MSs at higher loading percentages, which may hinder effective flow through the vascular system. This highlights the importance of optimizing Fe(CO)_5_ content in the formulation of PAM@Fe(CO)_5_ MSs to ensure both efficacy and safety in potential clinical applications.

Fe(CO)_5_, as a key component of our MSs, possesses several unique physicochemical properties that promise good prospects for application in MHT [28]. First, Fe(CO)_5_ exhibits a favorable magnetic heating effect, allowing it to generate heat effectively in AMF for MTA [28,29]. Additionally, the chemical stability and relatively low toxicity of Fe(CO)_5_ broaden its applications in the biomedical field. Compared to traditional magnetic nanomaterials, Fe(CO)_5_ MSs can remain stable in the in vivo environment and are less prone to degradation, which is significantly advantageous for long-term efficacy. Furthermore, the synthesis process of Fe(CO)_5_ is relatively simple and amenable to large-scale production, facilitating its clinical application [28,29].

In terms of characterization, thermogravimetric analysis (TGA) confirmed the actual content of Fe(CO)_5_ in the synthesized MSs (Fig. 1F). The X-ray diffraction (XRD) patterns clearly revealed the characteristic peak of PAM at 2θ = 21°, while Fe(CO)_5_ exhibited peaks at 2θ values of 44.8°, 65.0°, and 82.3°, corresponding to the (100), (220), and (111) crystal planes of Fe(CO)_5_, respectively (JCPDS No. 06–0696). These spectroscopic analyses confirmed the successful preparation of the PAM@Fe(CO)_5_ MSs (Fig. 1G). The hysteresis loop graph indicated that the PAM@Fe(CO)_5_ MSs exhibited zero remanence and coercivity, confirming the superparamagnetic nature of the magnetic material. Due to the non-magnetic coating layer of PAM@Fe(CO)_5_ MSs, the saturation magnetization of PAM@Fe(CO)_5_ MSs is lower compared to that of nano-sized Fe(CO)_5_ (Fig. 1H). The combination of nano-sized Fe(CO)_5_ and micron-sized PAM ensured that the magnetic material retains the advantages of ferromagnetic materials, which could exhibit high saturation magnetization under small external magnetic fields, while maintaining a condition where the remanent magnetization was zero after the external magnetic field was removed.

We subjected PAM@Fe(CO)_5_ MSs containing different Fe(CO)_5_ contents (5 %, 15 %, and 25 %) to testing in an AMF. Notably, the temperature changes of the PAM@Fe(CO)_5_ MSs under specific AMF strengths significantly depended on the Fe(CO)_5_ content. These results indicated that MSs containing 25 % Fe(CO)_5_ could rapidly heat to 85 °C under specific AMF conditions while maintaining stable magnetic thermal efficiency, providing theoretical support for their application in localized tumor treatment (Fig. 1I–K). Furthermore, under simulated long-term physiological conditions, PAM@Fe(CO)_5_ MSs exhibited excellent stability. No significant changes in particle size were observed after storing in PBS (pH 7.4, 10 mM) or 10 % FBS for 24 h, storing at 4 °C for 30 days, and 100-fold dilution in PBS (Fig. 1L). This stability may be attributed to the protective environment provided by the isotonic buffer and serum, which helps maintain the structural integrity of the particles over time and under varying conditions. Additionally, the low temperature during storage likely minimized any degradation processes that could affect particle size.

### 3.2 Cell experiments

**Fig. 2:**
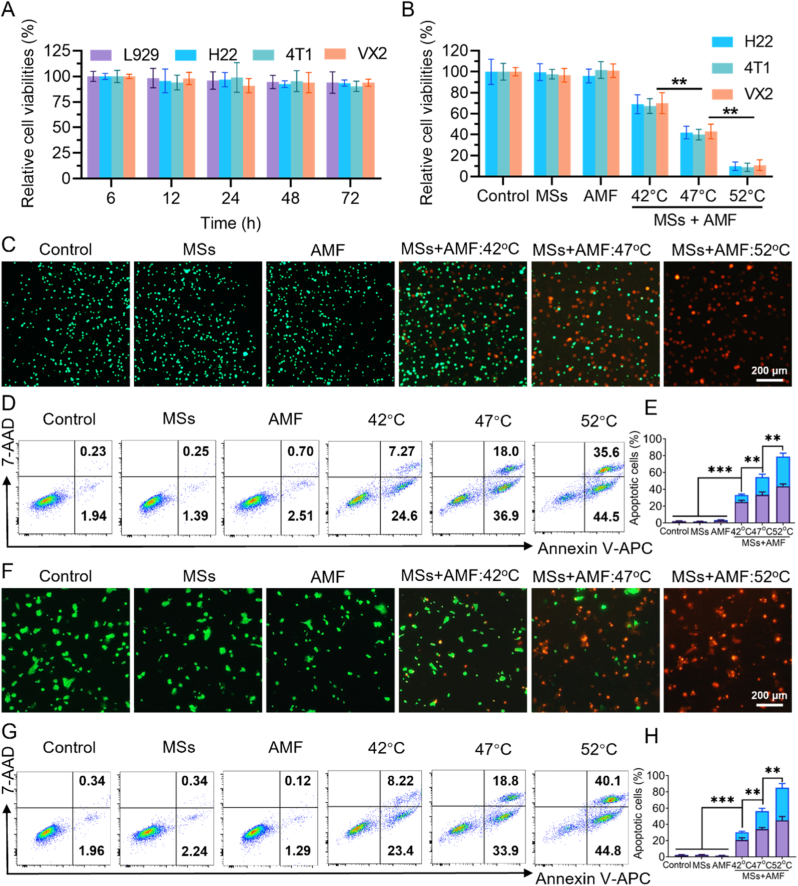
**In vitro experimental results of MTA using PAM@Fe(CO)_5_** **MSs**. (A) Relative viability of different cell types (L929, H22, 4T1, and VX2) after 72 h of treatment (n = 5). (B) Relative viability of different cancer cells (H22, 4T1, and VX2) after MHT using PAM@Fe(CO)_5_ MSs (n = 5). (C) Confocal fluorescence images showing H22 cells stained with Calcein AM (green, indicating live cells) and propidium iodide (PI, red, indicating dead cells) following various treatments. Scale bar = 100 μm. Evaluation of apoptosis in H22 (D) and 4T1 (F) cells using dual staining with Annexin V-APC/7-AAD. Quantitative analysis of H22 (E) and 4T1 (H) cells apoptosis. H22 mouse hepatocellular carcinoma cells, 4T1 mouse breast cancer cells, L929 mouse fibroblasts, and rabbit VX2 tumor cells. (For interpretation of the references to color in this figure legend, the reader is referred to the Web version of this article.)

For the in vivo applications, the biocompatibility of PAM@Fe(CO)_5_ MSs is a critical consideration. Therefore, we selected L929, H22, 4T1 and VX2 cells to evaluate the biocompatibility of MSs. MTT cytotoxicity assays were performed (Fig. 2A), and the results indicated that none of them demonstrated significant cytotoxicity, indicating that the MSs possess good biocompatibility. To further explore the cytotoxic effects of PAM@Fe(CO)_5_ MSs on tumor cells under AMF, H22 and 4T1 tumor cells were exposed to PAM@Fe(CO)_5_ MSs containing 25 % Fe(CO)_5_ (50 μm in diameter) while applying AMF for 5 min. The intensity of the AMF was calibrated to achieve temperatures of 42 °C, 47 °C, and 52 °C. About 60 % of the cells demonstrated cell death at 47 °C, and this figure increased to approximately 90 % at 52 °C (Fig. 2B).

To further validate the temperature-dependent toxicity of PAM@Fe(CO)_5_ MSs on H22 cells, fluorescence microscopy was employed to assess live (green) and dead (red) cells (Fig. 2C). No significant red fluorescence signals were detected in cells treated individually with either AMF or PAM@Fe(CO)_5_ MSs, indicating that PAM@Fe(CO)_5_ MSs exhibit good biocompatibility and low cytotoxicity. In contrast, in the group treated with both AMF and PAM@Fe(CO)_5_ MSs, cell death became increasingly evident as the temperature rose. These results demonstrate that the combination of MSs and AMF induces temperature-dependent cell death in H22 cells, with enhanced tumor cell killing efficacy at higher temperatures. Flow cytometry analysis also confirmed that no significant apoptosis was detected in cells treated individually with either AMF or PAM@Fe(CO)_5_ MSs, further supporting the notion that PAM@Fe(CO)_5_ MSs possess good biocompatibility and low cytotoxicity. In contrast, MHT mediated by PAM@Fe(CO)_5_ MSs significantly induced apoptosis in H22 cells, with apoptosis rates of 33.2 %, 54.6 %, and 78.9 % observed at temperatures of 42 °C, 47 °C, and 52 °C, respectively (Fig. 2D and E). A similar increase in MHT-induced apoptosis was noted in 4T1 cells (Fig. 2F and H). These findings underscore the good in vitro compatibility of PAM@Fe(CO)_5_ MSs and highlight the critical role of temperature control in enhancing the effectiveness of MHT.

### 3.3 In vivo MTA

Thermal ablation therapy has demonstrated its ability to effectively elicit anti-tumor immune responses [[30], [31], [32]]. In clinical practice, we found that the proportion of T cells in the thermal ablation group (microwave ablation, MWA) surgical specimens was significantly greater than that in the biopsy specimens (pre-MWA), indicating that thermal ablation therapy enhances the infiltration of T cells (Fig. S1). PD-L1 expression following thermal ablation was characterized, as this molecule plays a crucial role as a checkpoint that limits T-cell responses [33,34]. The PD-L1 proportion in surgical specimens from the thermal ablation group was significantly greater than that in biopsy samples, suggesting that MWA treatment increased PD-L1 expression, leading to immune suppression. These findings suggested that thermal ablation not only enhanced T cell immune responses in patients with HCC but also induced immune suppression. This dual effect created opportunities for the combination of thermal ablation with immune checkpoint inhibitors in the treatment of tumors.

**Fig. 3:**
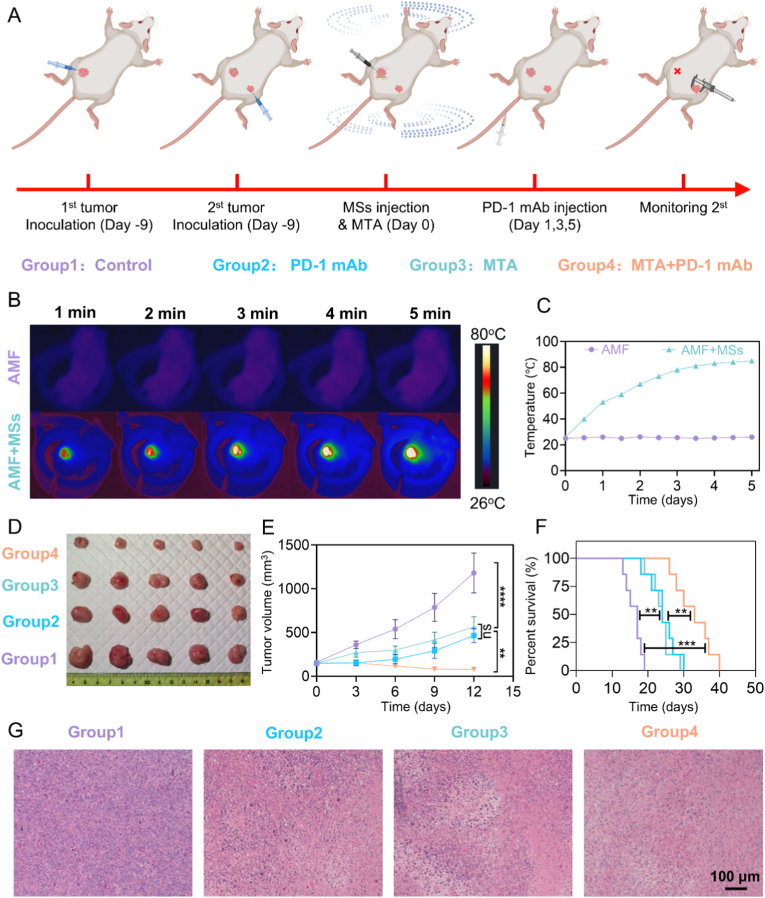
**In vivo experimental results of MTA using PAM@Fe(CO)_5_** **MSs**. (A) Schematic illustration of in vivo MTA treatment of H22 tumors using PAM@Fe(CO)_5_ MSs. (B&C) Infrared thermal images and temperature change curves induced by MTA. (D) Tumor sizes at 12 days post-MTA (n = 5). (E&F) Tumor growth and survival curves of H22 tumor-bearing mice following different treatments (n = 7). (G) Microscopic images of H&E-stained H22 tumor sections collected from each group.

Given the superior performance of PAM@Fe(CO)_5_ MSs in MTA, we constructed a bilateral subcutaneous tumor model in mice using H22 cells and conducted a series of in vivo animal experiments. The focus of the study was to evaluate the efficacy of in situ tumor ablation through MTA and the inhibitory effects of combining MTA with PD-1 mAb on distant tumors (Fig. 3A). Remarkably, in the presence of the AMF, the temperature of the injected PAM@Fe(CO)_5_ MSs in situ tumors increased rapidly to approximately 80 °C within about 5 min. In contrast, the group that was exposed only to the AMF did not exhibit any notable changes in temperature (Fig. 3B and C). The rapid increase in temperature of the PAM@Fe(CO)_5_ MSs in situ tumors under an AMF is due to the MSs’ superparamagnetic properties, which enable them to generate heat through magnetic hyperthermia while the AMF-only group showed no significant temperature change due to the absence of heat-generating material.

In the control group, distant metastatic tumors grew rapidly. After 12 days of treatment, the combined treatment group receiving both MTA and PD-1 mAb exhibited a significant decrease in the volume of distant metastatic tumors (Fig. 3D and E). Mice treated with this combination therapy exhibited the most substantial inhibition of metastatic tumors, along with a notable increase in survival time (Fig. 3F). H&E staining results further confirmed the favorable inhibitory effects of the combined treatment on distant metastatic tumors (Fig. 3G). These findings demonstrated the potential of combining MTA with immune checkpoint inhibitors, and future studies could explore the integration of other immunotherapeutic strategies with MTA.

H&E staining of tissue sections from the main organs in each treatment group revealed that the structure of the liver and spleen appeared largely intact, with no evident cellular damage or necrosis. The glomerular and tubular structures in the kidneys were maintained in all groups, showing no significant pathological alterations. Myocardial fibers were orderly arranged across all groups, and no signs of necrosis or inflammation were observed. The H&E images of the lungs from the control group, PD-1 mAb group, and MTA group mice indicated that the alveolar structure remained intact, with no significant inflammation or cell damage detected. In contrast, the H&E images from the MTA + PD-1 mAb group showed thickening of the alveolar walls and interalveolar septa, accompanied by inflammatory cell infiltration (Fig. S2). This finding may be attributed to insufficient aseptic techniques during the administration of the PD-1 mAb and the process of MTA, which could have led to lung infections in the mice rather than indicating significant toxicity. This observation underscores the importance of maintaining aseptic conditions. Throughout the treatment, all tumor-bearing mice exhibited stable vital signs, with no significant abnormalities observed. Overall, these experimental results suggest that the various treatment methods demonstrate good safety profiles and do not induce toxicity in major organs.

### 3.4 Effects on the immune response

**Fig. 4:**
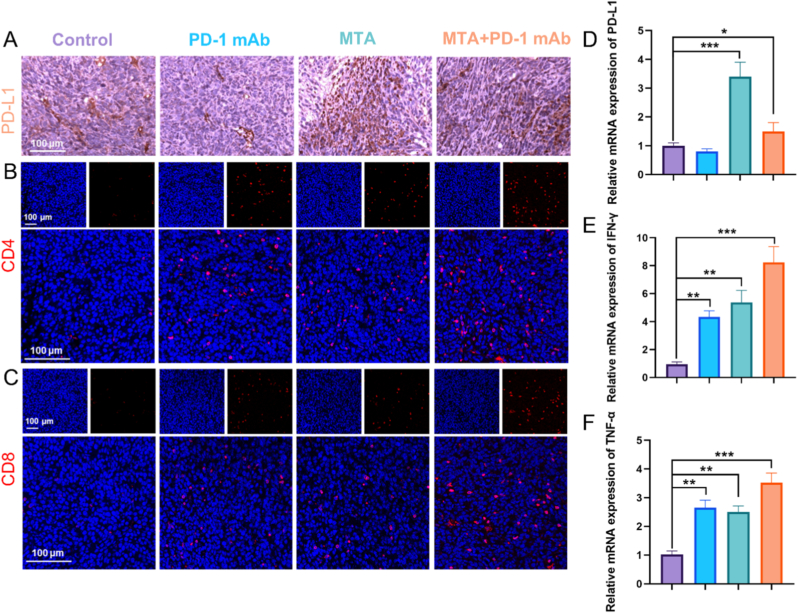
**Immune therapy induced by MTA combined with PD-1 mAb.** (A) Immunohistochemical staining images showing PD-L1 expression on the surface of H22 tumor cells. Immunofluorescence staining images show the infiltration of CD4^+^ T cells (B) and CD8^+^ T cells (C) within H22 tumors. Analysis of relative mRNA expression levels of PD-1 (D), IFN-γ (E), and TNF-α (F) in tumor tissues from each treatment group (n = 4).

Immunohistochemical examination of distant tumor showed that the MTA group demonstrated a marked increase in PD-L1 expression compared to the control group, suggesting that MTA treatment led to elevated levels of PD-L1. Although PD-L1 expression levels were already high in H22 tumor tissues, MTA treatment further increased these levels (Fig. 4A). The presence of PD-L1 inhibits the interaction between tumor-infiltrating lymphocytes and cancer cells, resulting in poor immune responses following MHT treatment alone. Under the stimulus of high PD-L1 expression after MTA, further blockade of the immune checkpoint PD-L1 may promote immune cell infiltration into the tumor, while subsequent PD-L1-targeting antibodies would enhance the immune effect. Immunofluorescence analysis indicated that there was a higher infiltration of T cells in tumor tissues following treatment with MTA + PD-1 mAb (Fig. 4B and C). MTA was utilized to treat in situ tumors. When combined with PD-1 mAb therapy, we observed an increase in CD4 and CD8 T cell infiltration in distant tumor tissues that did not receive MTA. This discovery indicates a systemic immune response rather than a local effect, underscoring the potential of MTA to induce immune changes at distant sites. PCR results also confirmed the significant upregulation of PD-L1 expression after MTA, providing favorable conditions for MTA combined with PD-1 mAb treatment of H22 tumors (Fig. 4D). Furthermore, IFN-γ and TNF-α are essential cytokines for the effector functions of T lymphocytes [[35], [36], [37]]. After the combined treatment, the mRNA levels of the two cytokines showed further increases (Fig. 4E and F). These results support the notion that MTA and PD-1 mAb synergistically enhance immune effector functions.

**Fig. 5:**
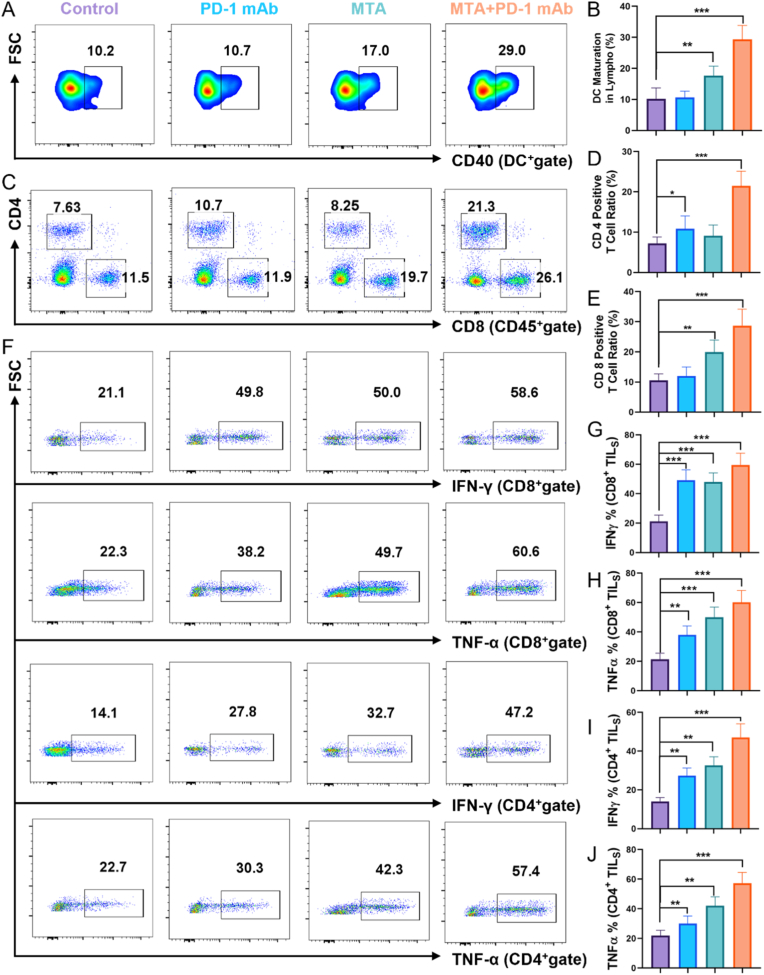
**Investigating the anti-tumor mechanism of MTA combined with PD-1 mAb.** (A&B) Representative flow cytometry plots and percentage statistics showing dendritic cell (DC) maturation in lymph nodes near distant metastatic tumors after different treatments. (C&D) Representative flow cytometry plots and percentage statistics of CD4^+^ and CD8^+^ T cells in tumors after different treatments. (E) Representative flow cytometry plots of CD8^+^IFN-γ^+^, CD8^+^ TNF-α^+^, CD4^+^ IFN-γ^+^, CD4^+^ TNF-α^+^ in distant metastatic tumors after different treatments. (F&G) Percentages of IFN-γ^+^ and TNF-α^+^ in CD8^+^ tumor-infiltrating T lymphocytes. (H&I) Percentages of IFN-γ^+^ and TNF-α^+^ in CD4^+^ tumor-infiltrating T lymphocytes (n = 4).

Flow cytometry was utilized to assess immune cells in metastatic tumors and adjacent lymph nodes. The findings revealed that mice in the MTA + PD-1 mAb group exhibited a notable increase in the maturation of dendritic cells (DCs) in the lymph nodes, reaching approximately 29.0 % (Fig. 5A and B, Fig. S3), significantly higher than that seen in the other groups. Mature DCs are essential for antigen presentation, which directly influences T cell activation. The enhanced maturation may occur through several pathways, including cytokine signaling and increased expression of co-stimulatory molecules (e.g., CD40), which are necessary for T cell priming. The presence of MTA may stimulate DCs directly or indirectly via dying tumor cells, enhancing their capacity to present tumor antigens. Additionally, after the combined treatment with MTA and PD-1 mAb, the proportion of T cells was markedly greater compared to the other groups (Fig. 5C–E, Fig. S4). Furthermore, the levels of the cytokines TNF-α and IFN-γ in the distant metastatic tumors were significantly higher in the two monotherapy groups compared to the control group. In contrast, the combination treatment notably boosted the effector functions of both CD4^+^ and CD8^+^ T cells (Fig. 5F–J, Fig. S4). IFN-γ, produced primarily by CD4^+^ T helper 1 (Th1) and CD8^+^ cytotoxic T cells, promotes a pro-inflammatory tumor microenvironment and enhances MHC class I expression on tumor cells, improving T cell recognition. TNF-α is involved in recruiting additional immune cells and enhancing the anti-tumor response [38,39]. Together, the increased expression of these cytokines indicates an activated immune state that favors tumor clearance. Overall, the combination of MTA and PD-1 mAb leverages multiple mechanisms of immune activation, including enhanced PD-L1 expression, increased immune cell infiltration, maturation of DCs, modulation of cytokine levels, and the induction of a systemic immune response. This multi-pronged approach not only improves treatment efficacy but also reprograms the immune landscape of the tumor microenvironment, providing a promising avenue for cancer immunotherapy.

### 3.5 Effectiveness and safety of MTA in VX2 orthotopic liver tumors in rabbits

**Fig. 6:**
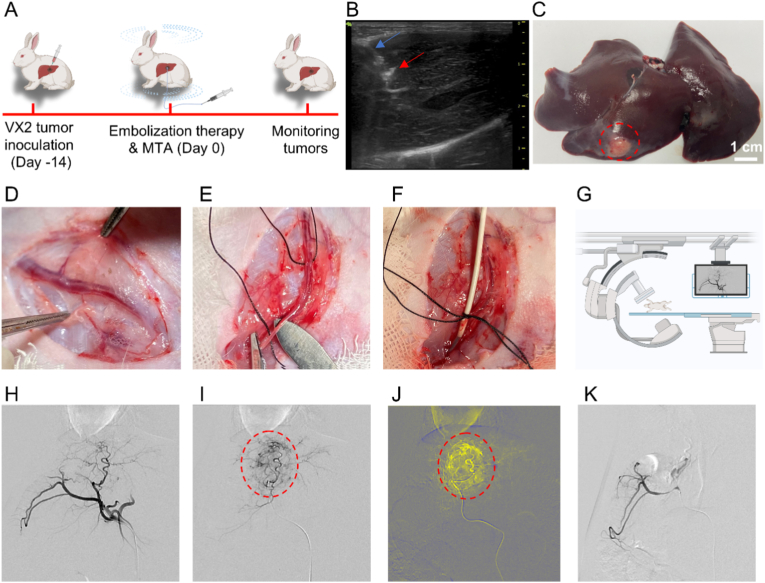
**Construction of rabbit VX2 orthotopic tumor model and MSs arterial embolization.** (A) Schematic of tumor-bearing and MTA. (B) Ultrasound-guided implantation of tumor tissue blocks, with blue arrows indicating the puncture needle and red arrows indicating the hyperechoic VX2 tissue blocks. (C) the VX2 tumor in the left lobe of the liver. (D–G) Femoral artery incision and super-selective catheterization under DSA guidance. (H) Angiographic image of the abdominal aorta demonstrating the celiac trunk and its branches. (I) Selective advancement of a microcatheter into the tumor-feeding artery, revealing abundant blood supply to the intrahepatic tumor with significant contrast enhancement (red circle). (J) False-color image illustrating the rich blood supply to the tumor (red circle). (K) Final angiogram confirming the absence of blood supply to the tumor after embolization. (For interpretation of the references to color in this figure legend, the reader is referred to the Web version of this article.)

MTA is not limited by the depth of penetration, and the magneto-thermal MSs are able to be uniformly distributed in the tumor tissue after the arterial embolization. This characteristic shows promise for overcoming the limitations of image-guided percutaneous puncture thermal ablation. To verify the feasibility and safety of PAM@Fe(CO)_5_ MSs-mediated MTA treatment for liver malignancies, we established a large animal rabbit orthotopic liver tumor model for preclinical evaluation. This research may provide references for the clinical application of TAE combined with MTA for the treatment of liver cancer (Fig. 6A).

TAE is performed under digital subtraction angiography (DSA) guidance, wherein a super-selective catheter is inserted into the branches of the hepatic artery, followed by the injection of embolic agents or drugs for liver cancer treatment [40,41]. With the aid of TAE technology, the application of drug-loaded MSs and radioactive MSs has gradually emerged in clinical settings in recent years, significantly improving the prognosis and quality of life for liver cancer patients [40,41]. This advancement has also motivated us to transition magnetic hyperthermia microspheres from basic research to clinical application. TAE is a critical step in MTA, allowing PAM@Fe(CO)_5_ MS to be precisely embolized into the supplying arteries of tumor, enhancing the effectiveness of MHT (Fig. 6A). In clinical practice, the ablation temperature for liver cancer typically ranges from 60 to 100 °C. This approach aims to rapidly increase the temperature of the tumor tissue while minimizing damage to the surrounding normal tissue. In reality, the ablation zone must extend at least 1 cm beyond the tumor margin to ensure complete coverage of the tumor tissue [42,43].

Under ultrasound guidance, VX2 tumor tissues were successfully implanted into the left lobe of the rabbits' livers. After approximately 12 days, CT scans confirmed the formation of tumors (Fig. 6B and C). Next, a microcatheter was introduced into the femoral artery, enabling DSA imaging that provided clear visualization of the arteries feeding the tumors (Fig. 6D–G). Angiographic image of the abdominal aorta revealed the celiac trunk and its branches (Fig. 6H). A microcatheter was selectively advanced into the tumor-feeding artery, which showed abundant blood supply to the intrahepatic tumor with significant contrast enhancement (Fig. 6I). The false-color image further demonstrated the rich blood supply to the tumor (Fig. 6J). TAE is a well-established technique for the treatment of liver cancer. After identifying the tumor-feeding arteries through DSA, small diameter MSs (50 μm) were slowly injected in a smooth, pulsed manner to occlude the tumor's blood supply effectively, utilizing the normal arterial blood flow to push the MSs into the feeding vessels, with complete occlusion as the endpoint. During the TAE procedure, real-time DSA imaging allowed for clear visualization of the tumor-feeding vessels, thereby minimizing the risk of ectopic embolization. The procedure was deemed complete when there was a significant reduction in perfusion or cessation of blood flow to the tumor-feeding arteries, indicating successful embolization [44]. Upon conclusion of the treatment, a final angiogram was performed to confirm the absence of blood supply to the tumor post-embolization (Fig. 6K). The use of contrast agents enabled physicians to monitor the treatment process in real-time under the guidance of DSA and facilitated the embolization of small-diameter MSs (50 μm) to the terminal branches of the tumor's blood supply arteries (Fig. S5). Additionally, the diameter of capillaries ranges from 4 to 12 μm, while MSs commonly used for TAE in clinical settings have diameters of 50–700 μm. MSs with a diameter of 50 μm can effectively occlude the terminal branches of the tumor's blood supply arteries without passing through the capillaries into the systemic circulation [[25], [26], [27]].Subsequently, the liver cancer rabbits were placed in an AMF, and infrared thermal imaging was used to monitor the temperature changes during AMF exposure. In vitro monitoring results indicated that the maximum temperature could reach approximately 70 °C (Fig. S6). We utilized in vitro real time infrared thermal imaging to monitor the temperature variations of tumors in tumor-bearing rabbits during MTA. This method not only allowed us to observe the temperature changes of tumors but also enabled us to assess the range of thermal damage induced by MTA.

Traditional embolization often leads to necrosis at the tumor center. However, the edges of the tumor may retain activity due to insufficient embolization, resulting in treatment failure. Clinically, it may be necessary to perform re-embolization or thermal ablation, which increases the treatment time, costs, and physical and psychological distress for patients. Thermal ablation typically requires a safety margin of at least 1 cm beyond the tumor edge to effectively eradicate the tumor and reduce the likelihood of recurrence. This requirement aligns with the purpose of using MSs embolization followed by MTA, as discussed in this paper. The impact of MTA is limited, which minimizes excessive damage to surrounding healthy tissues and reduces the risk of serious adverse consequences. The combination of MSs embolization followed by MTA achieves a therapeutic effect comparable to that of traditional TAE and thermal ablation, thereby improving the success rate of tumor treatment and reducing the possibility of tumor recurrence [45,46].

^18^F-FDG is a radiolabeled glucose used in PET imaging, widely applied due to its higher uptake in tumor cells. PET imaging provides significant advantages in animal experiments, including the ability to visualize metabolic and physiological processes in real-time. Its high sensitivity allows for the early detection of changes in tumor viability and therapeutic response, making it particularly effective for assessing the efficacy of treatments like magnetothermal microspheres. The standardized uptake values (SUVs) is a key parameter for quantifying ^18^F-FDG uptake, aiding in the assessment of tumor malignancy and treatment response [47,48].

**Fig. 7:**
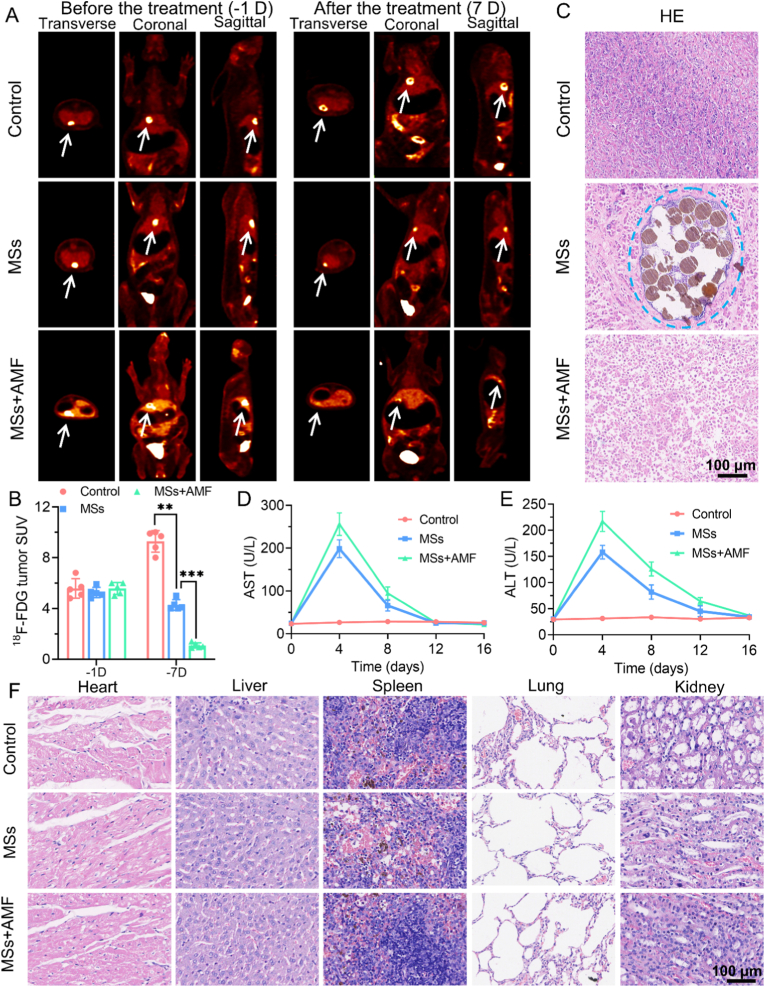
**In vivo therapeutic effects of MTA in a rabbit model of liver cancer.** (A) ^18^F-FDG PET images obtained before and after different treatment regimens, illustrating the metabolic activity of tumors in each treatment group. The images highlight changes in glucose metabolism, with decreased uptake indicating therapeutic efficacy. The white arrows indicate the location of the tumors. (B) Changes in standardized uptake values (SUVs) of tumors in different treatment groups (n = 5). (C) H&E staining images of tumors from each treatment group. The blue circle indicates PAM@Fe(CO)_5_ MSs. Scale bar = 100 μm. (D&E) Changes of alanine aminotransferase (ALT) and aspartate aminotransferase (AST) at different time points post-MTA in each group (n = 5). These data assessed liver function and the safety of the MTA. (F) H&E staining images of major organs from rabbits in different treatment groups. Scale bar = 100 μm. (For interpretation of the references to color in this figure legend, the reader is referred to the Web version of this article.)

To assess the therapeutic effects, we utilized ^18^F-FDG PET imaging (Fig. 7A and B). The PET imaging results showed pronounced signals in the HCC tissues prior to treatment, characterized by high SUVs. Following a week of treatment, the control group displayed heightened ^18^F-FDG signals as a result of tumor progression, while the regions receiving MSs embolization showed a relative decrease in signals. Significantly, the MSs + AMF group exhibited only weak ^18^F-FDG signals in the tumors, showing the magnetic thermal treatment successfully reduced tumor activity.

To further validate the therapeutic efficacy of MTA, H&E staining was used to analyze the tumor tissues. It could be found that the degree of necrosis in the tumor tissues of the MTA group was greater than the other groups, confirming the effectiveness of MTA treatment (Fig. 7C). Additionally, we assessed the safety of MTA treatment, revealing that both the MS embolization group and the MSs + AMF group experienced some degree of impact on liver function, with ALT and AST levels peaking on postoperative day 4 and returning to normal levels around postoperative day 12 (Fig. 7D and E). It is important to note that no toxicity was observed in key organs such as the heart, liver, and lungs during the safety assessment (Fig. 7F). The accumulation of inflammatory cells in the spleen may be attributed to the activation of the immune system, rather than indicating apparent toxicity. The structural disorder observed in the kidneys and the transient reduction in glomerular architecture could be related to the use of the contrast agent during TAE for tumor imaging, as the contrast agent requires metabolism by the kidneys. No significant changes were observed in serum cardiac and renal function indicators in the treated rabbits at different time points after treatment, and the differences between groups were not statistically significant (*P* > 0.05), further confirming that MTA has no toxicity on cardiac and renal function (Fig. S7). Throughout the treatment, the vital signs of all tumor-bearing rabbits remained stable, and no significant abnormalities were detected. These experimental results collectively demonstrate the good feasibility and safety of MTA treatment.

## 4 Conclusion

In summary, this study introduces an innovative approach for non-invasive MTA for treating HCC using magnetic hyperthermia MSs. The findings indicate that PAM@Fe(CO)_5_ MSs can effectively generate heat and kill tumor cells under the influence of AMF, while also activating the host's T-cell immune response, significantly enhancing anti-tumor efficacy. Particularly, when combined with immunotherapy, a synergistic effect was observed that enhanced the effector functions of immune cells and suppressed the growth of metastatic tumors. Furthermore, research conducted in large animals demonstrated that MTA avoids risks and tissue damage associated with traditional percutaneous ablation techniques, showcasing excellent safety and efficacy. This treatment approach is not limited to HCC, it could also be relevant for managing other solid tumors that are highly vascularized.

The combination of MTA with immunotherapy presents a promising strategy to enhance the efficacy of cancer treatment, particularly in HCC, by inducing local tumor destruction while simultaneously stimulating systemic anti-tumor immune responses. This synergistic approach not only targets and reduces tumor burden but could also disrupt the immune suppressive microenvironment, overcome resistance mechanisms and improving patient outcomes. This research offers fresh perspectives on HCC treatment, promoting a shift from traditional minimally invasive ablation methods to safer and more efficient non-invasive approaches, with significant clinical application prospects.

## CRediT authorship contribution statement

**Zepeng Yu:** Writing – original draft, Software, Methodology, Investigation, Funding acquisition, Formal analysis, Data curation, Conceptualization. **Yaping He:** Writing – original draft, Methodology, Investigation, Formal analysis, Data curation, Conceptualization. **Mengmeng Wang:** Methodology, Investigation. **Jiaofeng Shen:** Methodology, Investigation. **Di Wang:** Investigation. **Andong Yu:** Software. **Jun Gu:** Writing – review & editing, Visualization, Validation, Supervision, Resources. **Zhihui Hong:** Visualization, Validation. **Zhijun Pei:** Supervision, Resources. **Xingwei Sun:** Writing – review & editing, Visualization, Supervision, Funding acquisition.

## Declaration of competing interest

The authors declare that they have no known competing financial interests or personal relationships that could have appeared to influence the work reported in this paper.

## Data Availability

Data will be made available on request.
