# Supplementary material for: Enhanced magnetic thermal ablation combined with immunotherapy for hepatocellular carcinoma using engineering microspheres
Source: Mater Today Bio. 2025 Nov 27;35:102597. doi: 10.1016/j.mtbio.2025.102597 (PMC12719205; doi:10.1016/j.mtbio.2025.102597)
Supplement: Multimedia component 1 [file mmc1.docx]

**Enhanced Magnetic Thermal Ablation Combined with Immunotherapy for Hepatocellular Carcinoma Using engineering microspheres**

**ZePeng Yu^1, 2,＃^, YaPing He^3,＃^, Mengmeng Wang^4,＃^, JiaoFeng Shen^5,＃^, Di Wang^1^, AnDong Yu^1^, Jun Gu^2,*^,** **Zhihui Hong^6,*^, ZhiJun Pei^6,^*,** **XingWei Sun^1,*^**

^1^Department of Interventional Radiology, The Second Affiliated Hospital of Soochow University, Suzhou 215004, China

^2^Center for Medical Ultrasound, The Affiliated Suzhou Hospital of Nanjing Medical University, Suzhou Municipal Hospital, Gusu School, Nanjing Medical University, Suzhou 215001, Jiangsu Province, China

^3^Department of Radiotherapy & Oncology, The Second Affiliated Hospital of Soochow University, Suzhou 215004, China

^4^Department of Pharmacy, the Second Affiliated Hospital of Soochow University, Suzhou, 215004, China.

^5^Department of Oncology, The Second Affiliated Hospital of Soochow University, Suzhou 215004, China

^6^Department of Nuclear Medicine, The Second Affiliated Hospital of Soochow University，Suzhou 215004, China

**^＃^ These Authors contributed equally to this work.**

**Correspondence to:**

**Jun Gu**, Center for Medical Ultrasound, The Affiliated Suzhou Hospital of Nanjing Medical University, Suzhou Municipal Hospital, Gusu School, Nanjing Medical University, Suzhou 215001, Jiangsu Province, China. E-mail: fzbh2026@163.com.

**Zhihui Hong**, Department of Nuclear Medicine, The Second Affiliated Hospital of Soochow University，Suzhou 215004, China. E-mail: [hongzhihui515@126.com](mailto:hongzhihui515@126.com)

**ZhiJun Pei**, Department of Nuclear Medicine, The Second Affiliated Hospital of Soochow University，Suzhou 215004, China. E-mail: zjpei@suda.edu.cn

**Xingwei Sun**, Intervention Department, The Second Affiliated Hospital of Soochow University, Suzhou 215004, Jiangsu, China. E-mail: sdfeysxw@163.com.

**Supplementary Tables**

**Supplementary Table 1.** The catalogue numbers and manufacturers of all flow cytometry antibodies. They were obtained from Biolegend or Invitrogen and diluted 1:300 for cell staining.

| Reagents | Catalogue numbers | Manufacturers |
| --- | --- | --- |
| Live(7-AAD) | 420404 | Biolegend, USA |
| CD45-PE | 103105 | Biolegend, USA |
| CD3(AF700) | 100216 | Biolegend, USA |
| CD19(Percp-cy5.5) | 152406 | Biolegend, USA |
| MHC-Ⅱ(FITC) | 107606 | Biolegend, USA |
| CD11c(BV605) | 117334 | Biolegend, USA |
| CD40(APC-cy7) | 124638 | Biolegend, USA |
| CD8(SB702) | 67-0081-82 | Invitrogen, USA |
| CD4(BV605) | 100548 | Biolegend, USA |
| IFN γ(APC) | 505810 | Biolegend, USA |
| TNFα(FITC) | 506304 | Biolegend, USA |

**Supplementary Table 2.** The list of primers.

| GENE | Forward primer | Reverse primer |
| --- | --- | --- |
| TNF-α | GGACTAGCCAGGAGGGAGAACAG | GCCAGTGAGTGAAAGGGACAGAAC |
| IFN-γ | CTGGAGGAACTGGCAAAAGGATGG | GACGCTTATGTTGTTGCTGATGGC |
| PD-L1 | AAGCCTCAGCACAGCAACTTCAG | TGTAGTCCGCACCACCGTAGC |

**Supplementary Figures**


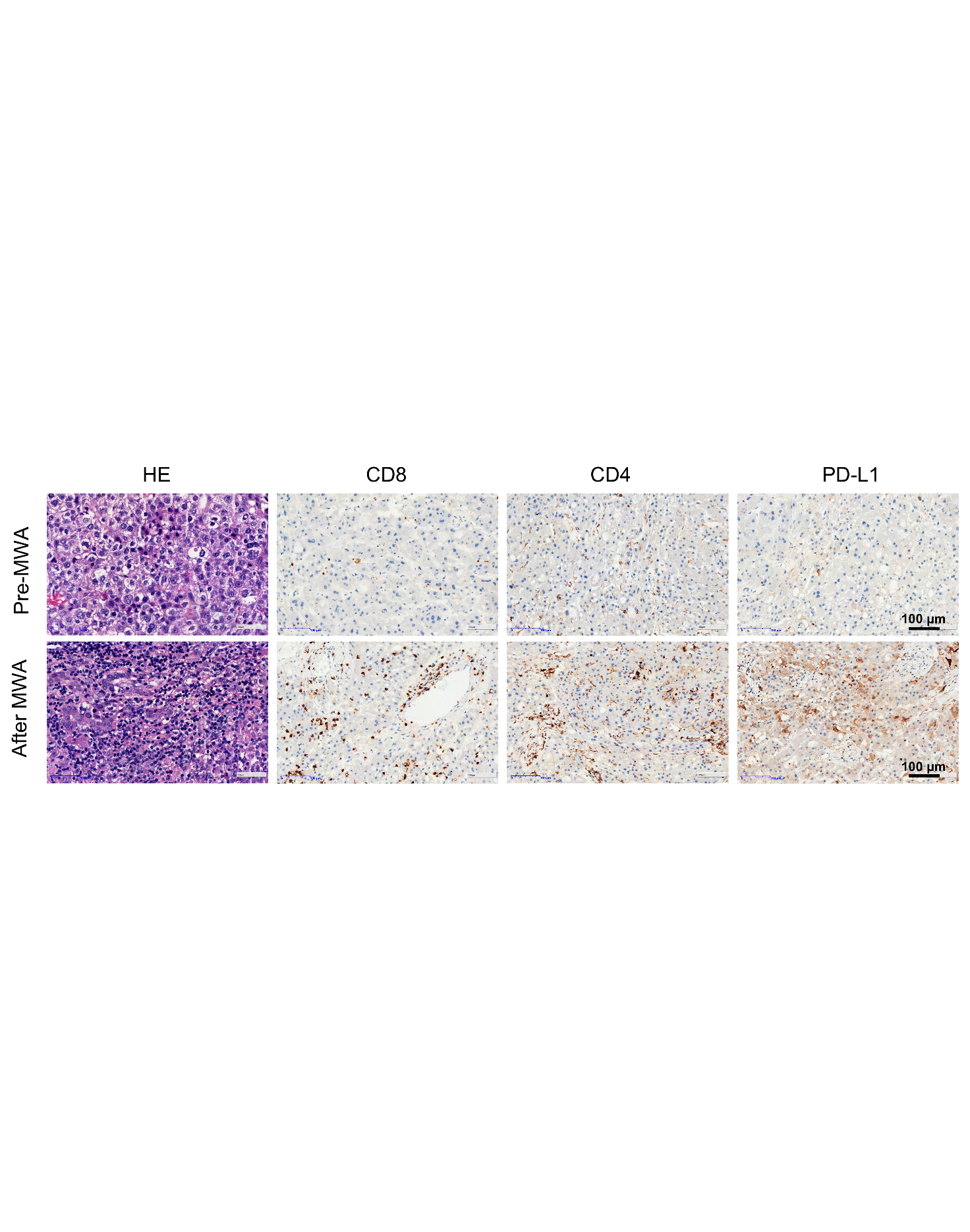


**Figure S1.** Representative microscopic images showing H&E, CD8, CD4, and PD-L1 staining results before and after microwave ablation (MWA) in the tumor microenvironment of liver cancer patients.


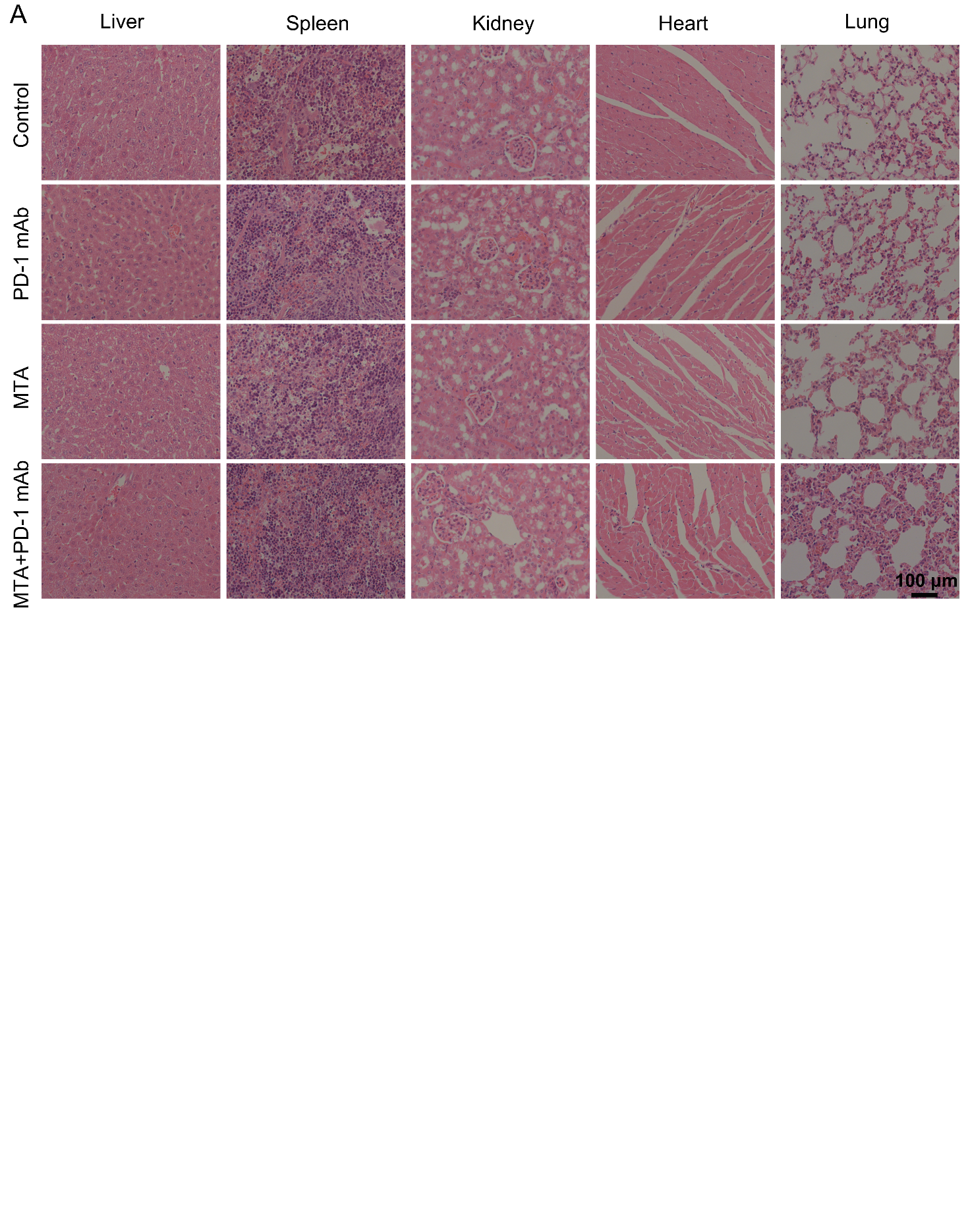


**Figure S2.** H&E staining images of major organs of mice in different treatment groups.


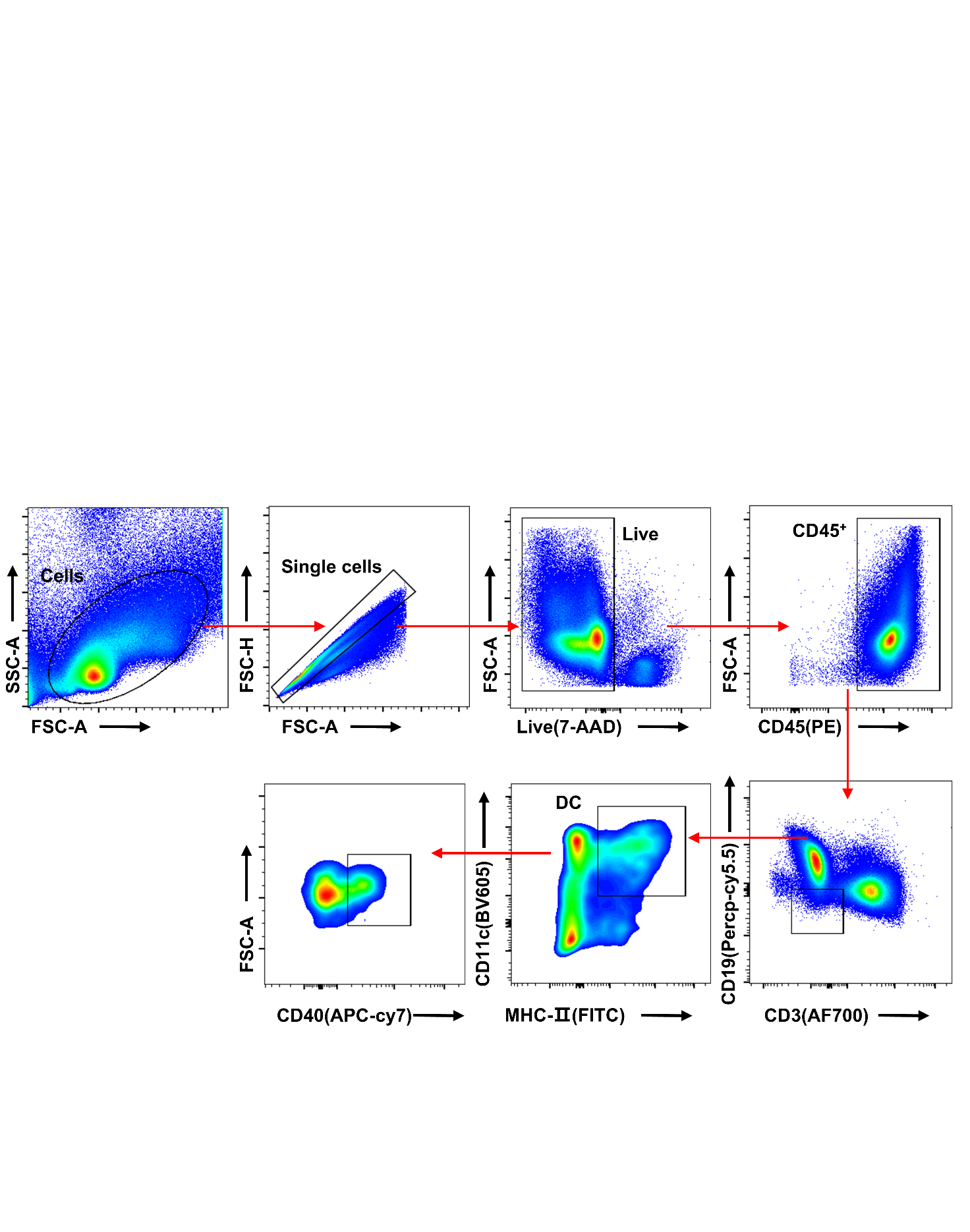


**Figure S3.** Gated strategy to determine the percentage of mature DCs in lymph nodes. CD3 (AF700) was used to label T cells, CD19 (Percp-cy5.5) was used to label B cells, and the CD3-CD19 double negative population removes T and B cells. Next, DCs were labeled with MHC-II (FITC) and CD11c (BV605). Finally, mature DCs were labeled with CD40 (APC-cy7).


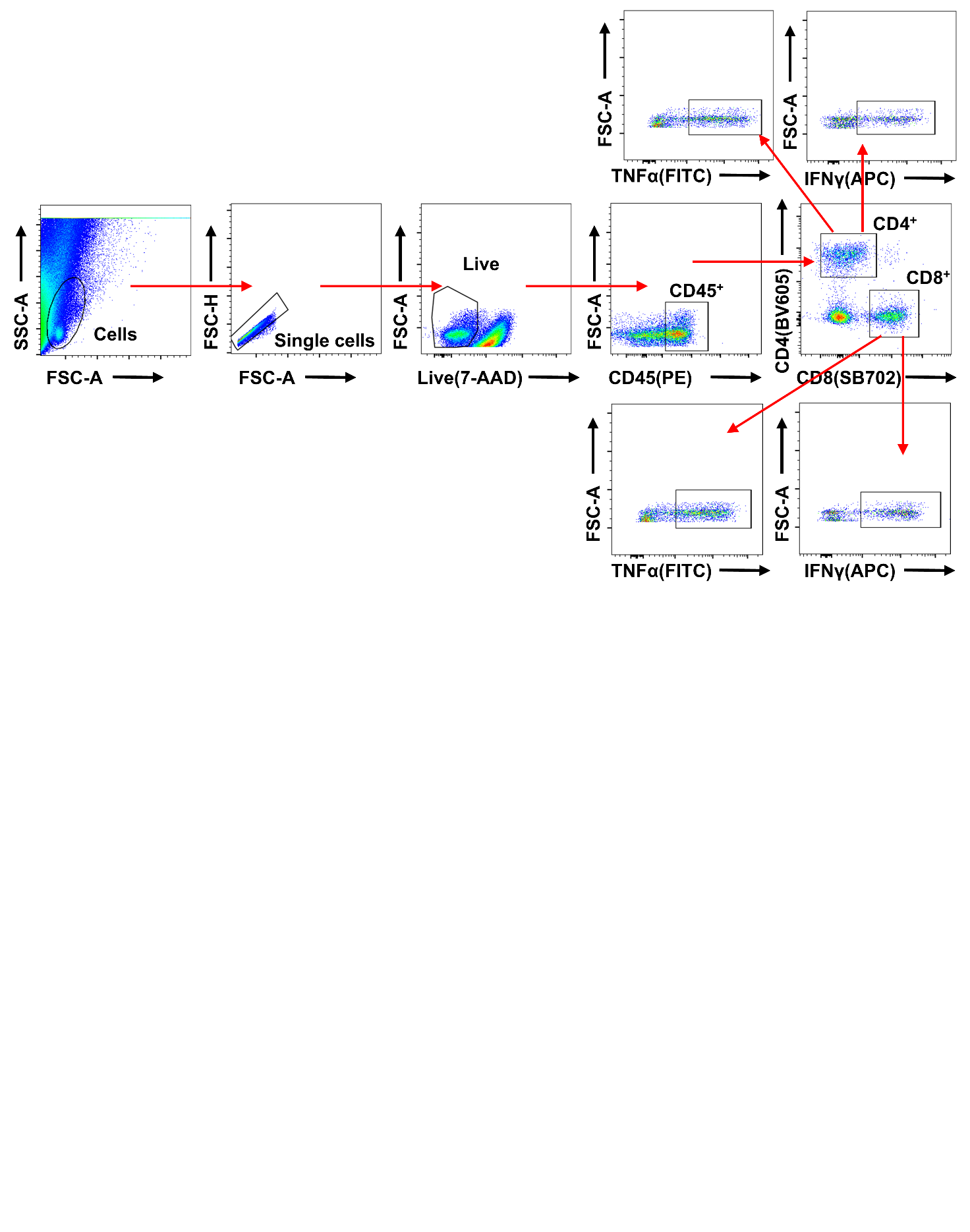


**Figure S4.** Gated strategy to determine the percentage of CD4^+^ and CD8^+^ T cells and their secreted cytokines TNF-α and IFN-γ in tumor tissue.


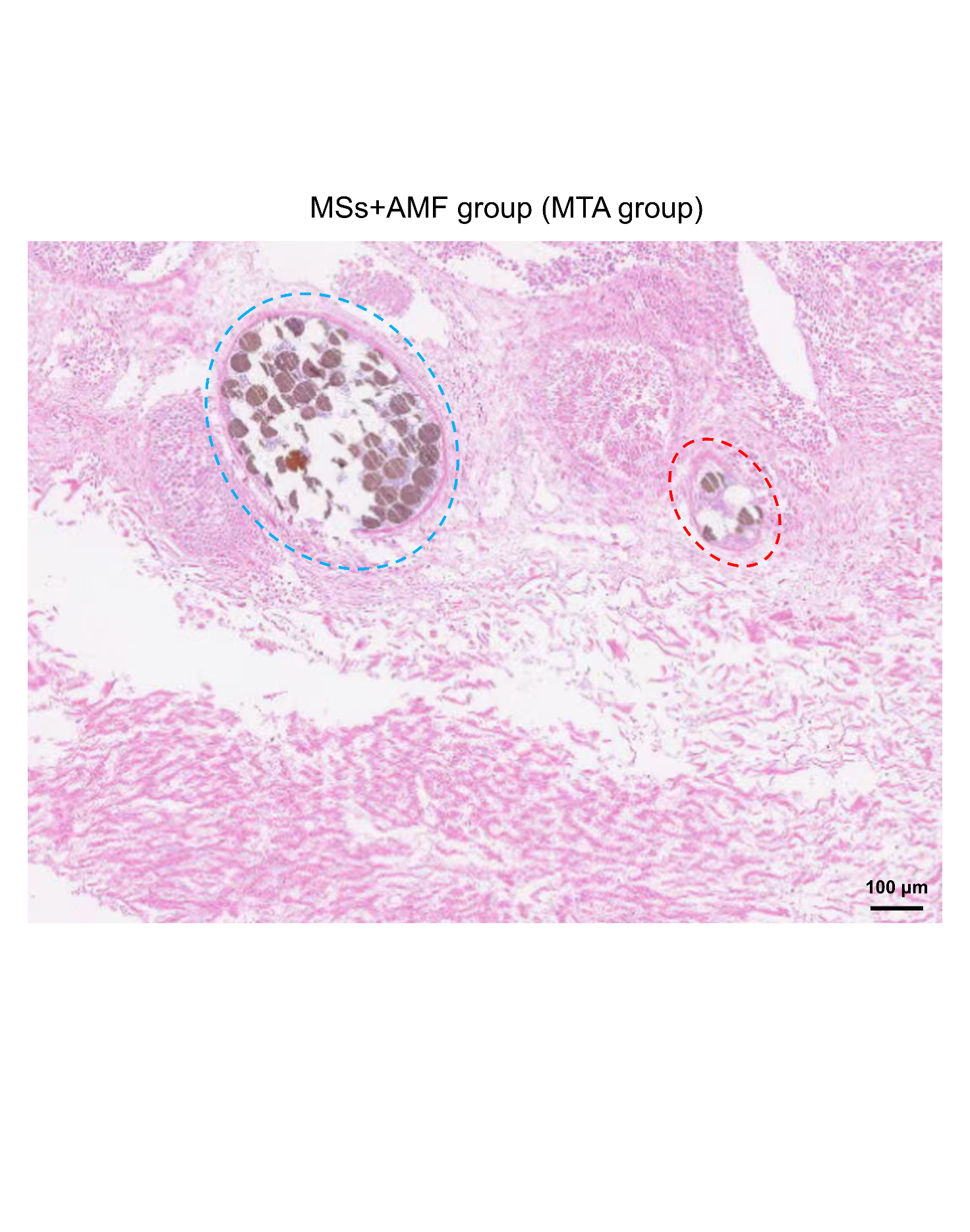


**Figure S5.** Following magnetic thermal ablation (MTA), tumor tissue necrosis is evident. The blue circle indicates the distribution of microspheres (MSs) within the tumor's blood supply arteries, while the red circle illustrates the embolization of MSs to the terminal branches of these arteries.


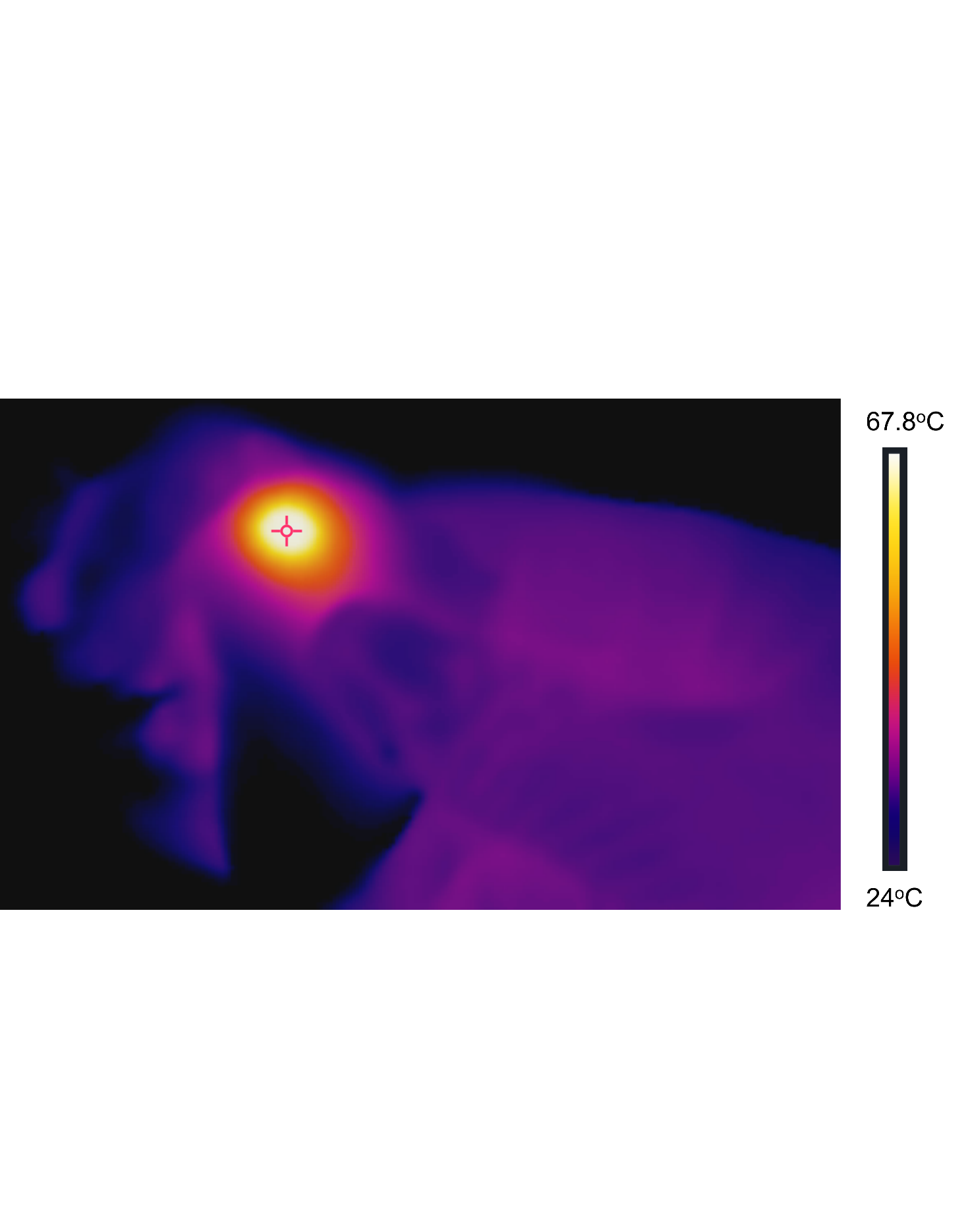


**Figure S6.** Infrared thermal images induced by MTA in the rabbit in situ liver cancer tumor model.


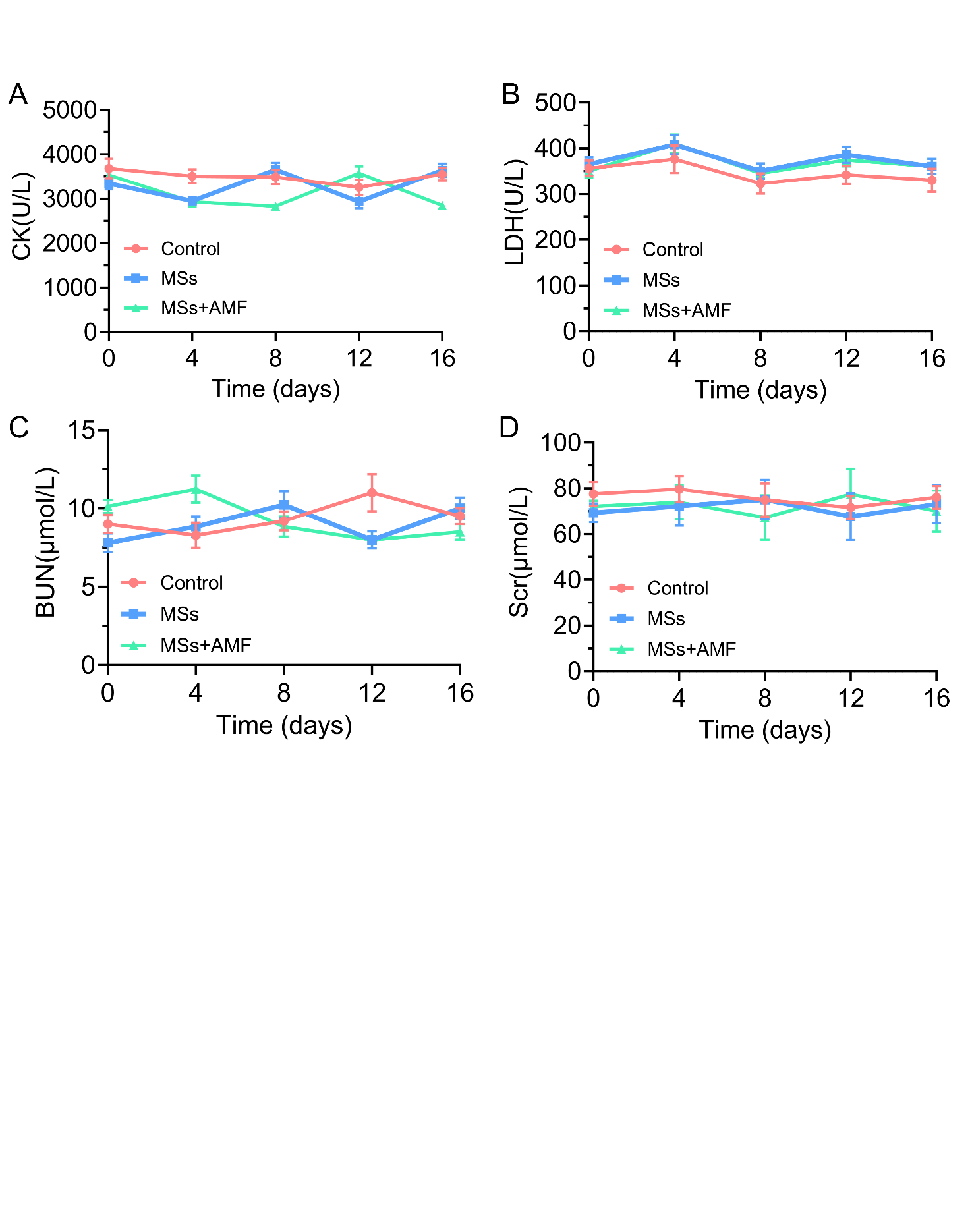


**Figure S7.** Changes in creatine kinase (CK), lactate dehydrogenase (LDH), BUN (blood urea nitrogen) and Scr (serum creatinine) levels after treatment in various group (n=5).
